# Can cardiovascular disease guidelines that advise treatment decisions based on absolute risk be improved?

**DOI:** 10.1186/s12872-016-0396-y

**Published:** 2016-11-15

**Authors:** Duncan J. Campbell

**Affiliations:** 1 Department of Molecular Cardiology, St. Vincent’s Institute of Medical Research, Fitzroy, VIC Australia; 2 The University of Melbourne, Parkville, VIC Australia

**Keywords:** Prediction, Statins, Absolute risk

## Abstract

**Background:**

Cardiovascular disease (CVD) will remain the predominant cause of death and a major cause of morbidity for the foreseeable future. Consequently, CVD prevention offers the greatest potential for the prevention of premature mortality and the compression of morbidity.

**Discussion:**

The 2013 guidelines of the American College of Cardiology and the American Heart Association expand the eligibility for CVD preventive treatment based on the calculated 10-year CVD risk derived from the pooled cohort equation to all persons who have a 10-year risk of CVD of ≥7.5% as estimated by the pooled cohort equation. Previous analyses show that the use of a uniform 10-year risk threshold of 7.5% for all ages disadvantages younger individuals for whom preventive therapy has most to offer. Here I show that reducing the threshold to 3% in younger adults (women aged <66 years and men aged <56 years) will substantially increase the number of cardiovascular events prevented at a similar number needed to treat to prevent one event. Importantly, this increase in cardiovascular event prevention will occur in individuals with greater life expectancy.

**Conclusion:**

Reducing the threshold 10-year risk of CVD derived from the pooled cohort equation for CVD preventive treatment to 3% in younger adults (women aged <66 years and men aged <56 years) will more effectively prevent premature mortality and compress morbidity to an older age.

## Background

Cardiovascular disease (CVD) will remain the predominant cause of death and a major cause of morbidity for the foreseeable future [1–3]. Consequently, CVD prevention offers the greatest potential for the prevention of premature mortality and the compression of morbidity [4]. Here, compression of morbidity refers to the prevention or postponement of cardiovascular events to a time closer to life expectancy, thereby restraining growth in the health costs of the aging population [4]. The 2013 guidelines of the American College of Cardiology and the American Heart Association (ACC-AHA) expand the eligibility for CVD preventive treatment based on the calculated 10-year CVD risk derived from the pooled cohort equation to all persons who have a 10-year risk of CVD of ≥7.5% as estimated by the pooled cohort equation [5, 6]. The pooled cohort equation calculates an individual's 10-year risk of first hard atherosclerotic CVD event (defined as first occurrence of nonfatal myocardial infarction, coronary heart disease death, or fatal or nonfatal stroke) [6]. There is concern, however, that a large proportion of younger adults destined to experience a cardiovascular event have an estimated 10-year risk that is below current thresholds for lipid reduction therapy [7]. Commenting on the failure of current guidelines to recommend lipid-lowering treatment for younger adults with low 10-year CVD risk but high lifetime risk, Leening et al. recently argued for a lifetime perspective in primary prevention of CVD based on lifetime risk [8]. Here, I present a case for lowering the 10-year risk threshold for preventive therapy in younger adults to achieve greater compression of morbidity with little additional cost per event prevented.

### Applying the pooled cohort equation to CVD prediction

**Fig. 1:**
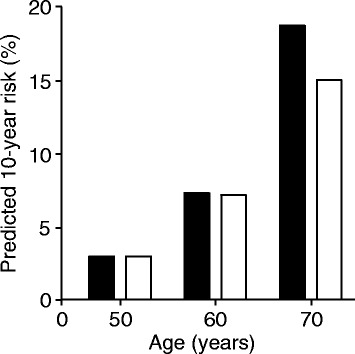
Impact of age on predicted 10-year risk of a cardiovascular event. For a woman (solid columns) with total cholesterol 213 mg/dl, HDL-C 50 mg/dl, untreated systolic BP 130 mmHg, nonsmoker, with diabetes, her estimated 10-year risk of a cardiovascular event is 3%, 7.2%, and 18.7% at ages 50, 60, and 70. For a man (open columns) with total cholesterol 213 mg/dl, HDL-C 50 mg/dl, untreated systolic BP 115 mmHg, nonsmoker, without diabetes, his estimated 10-year risk of a cardiovascular event is 3%, 7.3%, and 15.1% at ages 50, 60, and 70. Predicted 10-year risk was calculated using the pooled cohort equation [6]

The dominant predictor of any algorithm for CVD prediction is age [9], with the risk increasing by more than 2-fold for each additional decade from age 50 to 70 years in both women and men, even when all other risk factors remain constant (Fig. 1). Thus, age alone will cause an individual aged 50 with a 10-year risk of 3% to have a 7.5% risk at age 60 and at least 15% risk at age 70. Age also predicts the number of additional years of health that might be gained from CVD prevention, with younger individuals expecting more additional years of health from any CVD prevention strategy than older individuals. There is debate about how effectively the expanded indications for statin therapy for primary prevention of CVD will prevent CVD. In their analysis of the application of the ACC-AHA guidelines to the National Health and Nutrition Examination Surveys of 2005–2010 Pencina found these guidelines mostly increased statin eligibility for older adults who would not have future cardiovascular events [10]. Moreover, Navar-Boggan et al. describe how application of a 10-year fixed risk threshold of 7.5% to the Framingham Offspring cohort led to clinically significant variation in guideline treatment performance across age- and sex-specific groups, with decreased sensitivity for predicting future CVD events in women and younger adults, indicative of poor calibration of the prediction algorithm across the spectrum of age in women and men [11]. Navar-Boggan et al. concluded that cholesterol treatment recommendations could be improved by using age- and sex-specific CVD risk thresholds [11].

### Applying the pooled cohort equation to the Framingham offspring cohort

**Fig. 2:**
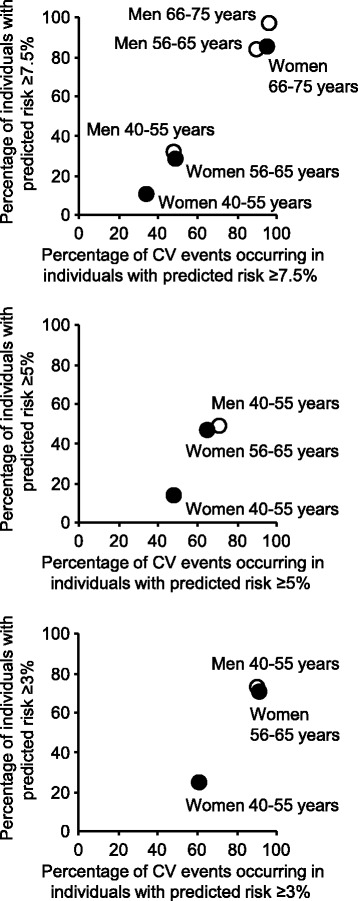
Relationship between the percentage of individuals with predicted 10-year risk ≥ threshold and the percentage of cardiovascular events occurring in these individuals, for different sex and age strata and for thresholds of 7.5%, 5% and 3%. Data were derived from the analysis of Navar-Boggan et al. [11]

Navar-Boggan et al. applied the pooled cohort equation to the CVD event data from the Framingham Offspring study, where the events were defined as a nonfatal myocardial infarction, death from coronary heart disease, fatal or nonfatal stroke, peripheral arterial disease, or heart failure [11]. They provide the percentage of Framingham Offspring cohort participants meeting the treatment threshold, and the sensitivity, specificity, positive predictive value and negative predictive value for recommendation of therapy to men and women aged 40–55, 56–65 and 66–75 years who had events over the subsequent 10 years, as determined from the pooled cohort equation. These data provide the proportion of each sex and age stratum that falls above the 10-year risk threshold of 7.5% and the proportion of events that occur in individuals above the threshold (Fig. 2). In order to achieve optimal prevention of CVD, it is necessary that the guidelines adequately capture individuals destined to experience CVD who can be offered preventive therapy. Figure 2 illustrates how a 10-year risk threshold of 7.5% captured 95% of women and 96% of men aged 66–75 years and 90% of men aged 56–65 years destined to have a cardiovascular event over the next 10 years. However, this threshold of 7.5% captured less than 50% of women and men aged 40–55 years and women aged 56–65 years destined to have a cardiovascular event.

The ACC-AHA guidelines recommend that lifetime risk estimation be used as a communication strategy for adults younger than 60 years who are free of atherosclerotic CVD and not candidates for lipid-lowering therapy, and state that it is reasonable to offer treatment with a moderate intensity statin to adults without CVD and diabetes who have a 10-year CVD risk of 5–7.5% [5]. Navar-Boggan et al. discuss how reducing the threshold risk from 7.5 to 5% improved sensitivity and specificity for men and women aged less than 66 years [11], but a threshold of 5% still failed to capture many women aged 56–66 years and women and men aged 40–55 years destined to have a cardiovascular event (Fig. 2). By contrast, a reduction in threshold to 3% resulted in a greater proportion of younger adults above the 3% threshold and at the same time enabled the capture of a greater proportion of those destined to experience a cardiovascular event. Reducing the threshold from 7.5 to 3% increased the proportion of women aged 40–55 years above the threshold from 11 to 25% and increased the proportion of events occurring in those above the threshold from 36 to 61% (Fig. 2). Similarly, the proportion of men aged 40–55 years above the threshold increased from 32 to 73% and the proportion of events occurring in those above the threshold increased from 48 to 90%. Moreover, the proportion of women aged 56–65 years above the threshold increased from 29 to 73% and the proportion of events occurring in those above the threshold increased from 49 to 91%. Furthermore, reduction of the threshold from 7.5 to 3% reduced the 10-year event rate in people below the threshold from 5 to 4% for women aged 40–55 years, from 14 to 7% for men aged 40–55 years, and from 8 to 4% for women aged 56–65 years. These event rates from the Framingham Offspring cohort are higher than predicted by the pooled cohort equation for a 3% CVD risk threshold because the pooled cohort equation does not use age- and sex-specific CVD risk thresholds [11].

Reducing the threshold risk for eligibility necessarily increases the number of individuals who become eligible for lipid-lowering therapy, with concern that treating more people with statins for longer (because they are younger) will increase the number who experience an adverse event from therapy. However, the risk of adverse events needs to be balanced against the potential benefits of therapy. Pandya et al. showed that lowering the 10-year threshold from 7.5% to as low as 3% would avert more atherosclerotic cardiovascular events cost effectively, although they failed to take account of the decreased sensitivity of the pooled cohort equation for predicting future CVD events in women and younger adults [12]. Limiting the lower threshold to women aged <66 years and men aged <56 years is likely to be even more cost effective because it would produce little change in the proportion of individuals above the threshold predicted to have a cardiovascular event, and thus would have little effect on the cost per event prevented. For a threshold of 7.5% in comparison with 3%, the proportion of individuals above the threshold predicted to have a cardiovascular event during the next 10 years was 23% and 18% for women aged 40–55 years, 28% and 23% for men aged 40–55 years, and 19% and 14% for women aged 56–65 years, respectively. The predicted number needed to treat for 10 years to prevent one cardiovascular event can be calculated from the proportion of individuals predicted to have an event and the percentage reduction in event rate from therapy. Assuming that lipid-lowering therapy reduces the CVD event rate by 25% [13, 14], for a threshold of 7.5% in comparison with 3%, the number needed to treat is 17.4 and 22.2 for women aged 40–55 years, 14.3 and 17.4 for men aged 40–55 years, and 21.1 and 28.6 for women aged 56–65 years. Importantly, a threshold of 3% would result in statin therapy being offered to individuals who would otherwise experience an event while waiting for their risk to reach the threshold of 7.5%. Thus, for 1000 women aged 40–55 years, an additional 20 women destined to experience an event over the next 10 years would be offered statin therapy, and for 1000 men aged 40–55 years, an additional 78 men destined to experience an event over the next 10 years would be offered statin therapy. Moreover, for 1000 women aged 56–65 years, an additional 47 women destined to experience an event over the next 10 years would be offered statin therapy.

If one assumes that lowering the threshold risk for eligibility does not change the cost per individual treated, then the similar numbers needed to treat for threshold risks of 7.5% and 3% for women aged <66 years and men aged < 56 years indicate similar cost effectiveness. Importantly, the lower threshold risk threshold is likely to be more cost effective because starting statin therapy at a younger, rather than older, age is predicted to result in more life years gained at lower cost per life year gained [15]. Although adherence to prescribed therapy for primary prevention is often poor and results in reduced efficacy and increased costs of such approaches, Helin-Salmivaara et al. reported that persons aged 45–74 years are more likely to continue statin use than younger or older age groups [16].

Previous attempts to improve treatment decisions based on 5- and 10-year risk have included the estimation of 30-year and lifetime risk [1–3, 17], with the intention to identify individuals of younger age with higher 30-year or lifetime risk who might benefit from preventive therapy. Such a strategy might lead to offering preventive therapy to a greater proportion of younger individuals destined to experience a cardiovascular event within 10 years. However, it is not known what 30-year or lifetime risk threshold should guide decisions or how effectively such a strategy would capture individuals aged 40–55 years who are destined to have a cardiovascular event well before their predicted life-expectancy. Importantly, treatment guidelines based on 30-year and lifetime risk do not incorporate an adjustment for the greater potential gain in years free of a cardiovascular event for younger individuals offered CVD preventive therapy. Compression of morbidity and reduction in health care costs are best achieved by CVD prevention in younger individuals with greater potential years of life free of CVD, not elderly individuals approaching the end of their life who may have not only a high CVD risk but also a high risk of death from non-cardiovascular causes. The present proposal to use a 10-year predicted risk threshold of 3% applies only to women and men aged 40–55 years and women aged 56–65 years. The threshold of 7.5% is quite satisfactory for women and men aged 66–75 years and men aged 56–65 years (Fig. 2). The increased number of younger adults offered CVD preventive therapy resulting from a reduction in risk threshold to 3% might be counter-balanced by the withdrawal of CVD preventive therapy from individuals approaching the end of life, for whom polypharmacy may produce more harm than good [18], and for whom the potential for CVD preventive therapy to extend life free of morbidity is small, such as for nursing home residents [19]. In a pragmatic randomized clinical trial Kutner et al. recently reported that withdrawal of statin therapy in adults with limited life expectancy is safe and may be associated with benefits including improved quality of life, use of fewer nonstatin medications, and a corresponding reduction in medication costs [20].

There are several arguments supporting a lower threshold 10-year predicted risk for prescribing lipid-lowering therapy in younger adults. Firstly, the high prevalence of obesity is accompanied by an increased cardiovascular risk in younger adults [21]. Secondly, younger adults are likely to gain more years of health from prevention of cardiovascular events than older adults, leading to the compression of morbidity and reduced heath care costs. Thirdly, in addition to lower mortality, morbidity and better quality of life in older age, improved cardiovascular health in middle age predicts reduced incidence of cancer, end-stage renal disease and dementia, further contributing to compression of morbidity [22–27]. Lastly, as discussed, lowering the threshold for younger adults prevents many more cardiovascular events in younger adults at a similar cost per event prevented as achieved by a threshold of 7.5%. Atherosclerotic disease develops over decades and these arguments for a lower risk threshold for CVD preventive therapy in younger adults are supported by evidence for the long-term impact of risk factors such as blood pressure and cholesterol levels on cardiovascular risk [8, 28, 29].

### Limitations

This analysis has limitations. It is based on historical data from the Framingham Offspring Study and may not be representative of contemporary communities. Moreover, the event data from the Framingham Offspring Study included peripheral arterial disease and heart failure, which are not included in the pooled cohort equation [6, 11]. However, the Framingham Offspring Study contributed to the derivation cohort used for the pooled cohort equation [6], and similar discrimination by the ACC-AHA guidelines against younger individuals at CVD risk was reported for the National Health and Nutrition Examination Surveys [10]. Older age and male sex are well-recognized risk factors for cardiovascular events and the differences in performance of the pooled cohort equation in women and men of different age strata are likely to be reflected in other communities. It has been argued that the pooled cohort equation over-estimates risk [30]. Nevertheless, despite possible inaccuracies in the predicted risk, the events recorded from the Framingham Offspring Study are real. It is, however, acknowledged that any risk algorithm requires calibration in the population to which it will be applied and, as demonstrated by Navar-Boggan et al., treatment recommendations could be improved by using age- and sex-specific CVD risk thresholds [11]. There is no such thing as a perfect threshold, and there will always be a trade-off between sensitivity for predicting events and the number above the threshold for whom treatment is recommended. It is necessary to balance the costs of intervention and potential harm from the therapy against the benefits of prevention and postponement of cardiovascular events. Moreover, there is uncertainty about the impact of any guidelines, and randomized trials may be necessary to determine which of different guidelines has maximum impact on CVD event rate and produces greatest compression of morbidity [31].

## Conclusions

The use of a uniform 10-year risk threshold of 7.5% for all ages disadvantages younger individuals for whom preventive therapy has most to offer [10, 11]. Reducing the threshold to 3% in younger adults (women aged <66 years and men aged <56 years) will substantially increase the number of cardiovascular events prevented at a similar cost per event prevented. Importantly, this increase in cardiovascular event prevention will predominantly occur in younger individuals in whom the prevention of events will compress morbidity to an older age. Although this analysis is focused on the ACC-AHA guidelines for lipid-lowering therapy, these arguments apply equally to blood pressure-lowering and other CVD preventive strategies.

## Abbreviations

ACC-AHA: American College of Cardiology and the American Heart Association
CVD: Cardiovascular disease
